# Longitudinal gut virome analysis identifies specific viral signatures that precede necrotizing enterocolitis onset in preterm infants

**DOI:** 10.1038/s41564-022-01096-x

**Published:** 2022-04-21

**Authors:** Emily A. Kaelin, Cynthia Rodriguez, Carla Hall-Moore, Julie A. Hoffmann, Laura A. Linneman, I. Malick Ndao, Barbara B. Warner, Phillip I. Tarr, Lori R. Holtz, Efrem S. Lim

**Affiliations:** 1 School of Life Sciences, Arizona State University, Tempe, AZ USA; 2 Center for Fundamental and Applied Microbiomics, Biodesign Institute, Arizona State University, Tempe, AZ USA; 3 Department of Pediatrics, Washington University School of Medicine, St Louis, MO USA; 4 Department of Molecular Microbiology, Washington University School of Medicine, St Louis, MO USA

**Keywords:** Gastrointestinal diseases, Microbiome

## Abstract

Necrotizing enterocolitis (NEC) is a serious consequence of preterm birth and is often associated with gut bacterial microbiome alterations. However, little is known about the development of the gut virome in preterm infants, or its role in NEC. Here, using metagenomic sequencing, we characterized the DNA gut virome of 9 preterm infants who developed NEC and 14 gestational age-matched preterm infants who did not. Infants were sampled longitudinally before NEC onset over the first 11 weeks of life. We observed substantial interindividual variation in the gut virome between unrelated preterm infants, while intraindividual variation over time was significantly less. We identified viral and bacterial signatures in the gut that preceded NEC onset. Specifically, we observed a convergence towards reduced viral beta diversity over the 10 d before NEC onset, which was driven by specific viral signatures and accompanied by specific viral-bacterial interactions. Our results indicate that bacterial and viral perturbations precede the sudden onset of NEC. These findings suggest that early life virome signatures in preterm infants may be implicated in NEC.

## Abstract (web summary)

Gut virome analysis of preterm infants at risk of developing necrotizing enterocolitis identifies a conserved viral signature that precedes disease onset.

## Main

Necrotizing enterocolitis (NEC) is a serious and sudden necroinflammatory complication of preterm birth^1^. NEC incidence in infants born at <32 weeks’ gestation ranges from 2 to 7% in high-income countries, with case mortality ranging from 22 to 38%^2^. NEC survivors face lifelong sequelae, including short bowel syndrome and neurodevelopmental disabilities^1^. The aetiology of NEC is unclear but risk factors in addition to preterm birth include formula feeding and prolonged use of antibiotics early in life^3^. Numerous studies suggest that gut microbiome alterations contribute to the development of NEC^4–6^, with several recent large studies converging on a risk community state consisting of over-representation of Gram-negative facultative bacteria (for example, *Gammaproteobacteria*, *Proteobacteria*) and relative under-representation of obligate anaerobic bacteria^4,6^. Notably, however, no single bacterial genus, species, serotype or sequence type has reproducibly been implicated as the cause of NEC. How the microbiome contributes to NEC pathogenesis is unclear but proposed mechanisms include stimulation of Toll-like receptor 4 by lipopolysaccharide from Gram-negative bacteria, leading to poorly controlled inflammatory responses in the preterm gut^1,7,8^. Some reports have associated eukaryotic viruses with NEC^9–13^ and a recent next-generation sequencing (NGS) study described a limited spectrum of bacteriophages, including enrichment of *Staphylococcus* phage 363_30, before NEC in preterm infants^5^, but these studies did not address overall virome composition and dynamics.

Factors such as breastfeeding, delivery route, antibiotics and the environment influence gut bacterial community composition and microbiome maturation^14,15^. Time series studies of the preterm gut microbiome identified choreographed patterns of microbiome acquisition^16,17^. For example, stool samples from preterm infants in the first days of life are characterized by high proportions of *Bacilli*, giving way over time to *Gammaproteobacteria* and then *Clostridia*^16^.

In contrast to the relatively stable adult virome^18,19^, a small number of studies have been performed, which suggest that temporal changes are common in the gut viromes of infants and young children, including changes in bacteriophage diversity and increases in prevalence and richness of eukaryotic viruses over time^20–22^. Bacteriophages are believed to influence gut bacterial communities^23,24^. Recent experimental evidence demonstrated that bacteriophages influence microbiome composition in mice^25^ and affect the microbiome and intestinal health after fecal microbiota transfer^26,27^. Moreover, virome alterations have been associated with inflammatory bowel disease and colitis, suggesting that the virome plays a role in digestive disorders^28–30^. Given the importance of microbiome acquisition and of the interacting role played by the virome in health and disease, it is logical to study the development of the preterm virome over time to understand factors that may influence health and disease.

In this study, we present a longitudinal, metagenomic NGS study of the gut viromes of 23 preterm infants. This cohort includes 9 infants who subsequently developed NEC and 14 controls matched for gestational age at birth and birthweight. We found substantial interpersonal variation in gut viromes across both phage and eukaryotic viruses at various ages in infants at risk for NEC. However, the viromes of infants who developed NEC converged towards a reduced level of beta diversity before NEC ensued and this convergence was characterized by specific viral signatures.

## Results

### Preterm virome varies within and between infants over time

**Extended Data Fig. 1:**
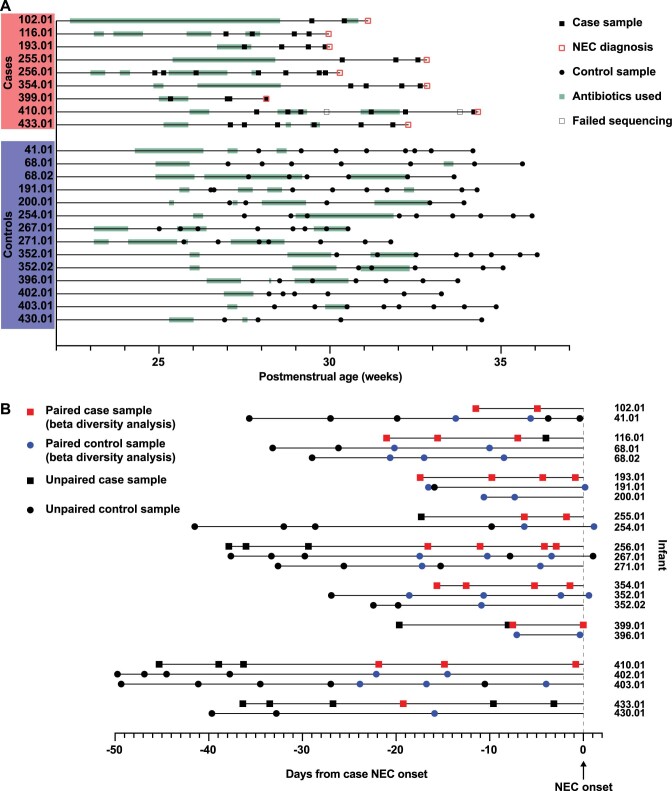
Sample timeline. **a**: All samples by postmenstrual age at sample collection. **b**: Samples used for analysis of time relative to NEC onset (Figs. 3 and 4). Matched case and control infants are grouped together. Paired samples used for beta diversity analysis are indicated in red and blue (Figs. 3a and 4b).

We analysed 138 stool samples collected over time from 23 preterm infants in the neonatal intensive care unit (NICU) at St. Louis Children’s Hospital (Supplementary Tables 1 and 2). Nine of the infants (cases) developed NEC and 14 infants matched for weight and gestational age at birth (controls) did not. Cases resembled controls in terms of sex and delivery route (Table 1). Postmenstrual age (PMA) (defined as weeks of gestation at birth plus postnatal age) at sample collection ranged between 24.9 and 34.2 weeks for case infants and 25.0 and 36.1 weeks for control infants (Extended Data Fig. 1a). Day of life at sample collection ranged from 6.5 to 75.1 for control infants and 2.4 to 58.2 for case infants. Two samples with sparse reads (6 and 29) were excluded from the analysis. We analysed a median of 412,905 (interquartile range (IQR) = 288,727–521,394) quality-filtered reads per sample (Supplementary Table 3). A total of 778,612 contigs were assembled from the infant stool samples (Supplementary Table 4), of which 40,210 were identified as viral.

**Table 1:** Cohort characteristics

Variable	Controls *n* = 14	Cases *n* = 9	Statistical significance
Gestational age at birth, weeks	25.5 (24.9–26.0)	25.0 (23.1–25.4)	NS, *P* = 0.19
Birth weight, g	810 (670–920)	780 (570–955)	NS, *P* = 0.82
Vaginal delivery	2 (14%)	4 (44%)	NS, *P* = 0.16
Male	5 (36%)	6 (67%)	NS, *P* = 0.21
1 and 5 min Apgar scores	2 (1–6) and 5.5 (2.8–6.3)	3 (1.5–5) and 5 (4.5–6)	NS, *P* = 0.93/NS, *P* = 0.96
Exposed to human milk during sampling period (yes)	13 (93%)	8 (89%)	NS, *P* > 0.99
Percentage of days of antibiotic exposure during the sampling period	25.3% (6.1–33.3%)	10.5% (0–29.4%)	NS, *P* = 0.25
Stool samples analysed per infant	7 (6–8)	4 (4–6)	*P* = 0.01

Statistical significance assessed by two-sided Mann–Whitney *U*-test for continuous variables and two-sided Fisher’s exact test for categorical variables. NS, not significant. Data are expressed as the median (IQR) or number (percentage) as appropriate.

**Fig. 1:**
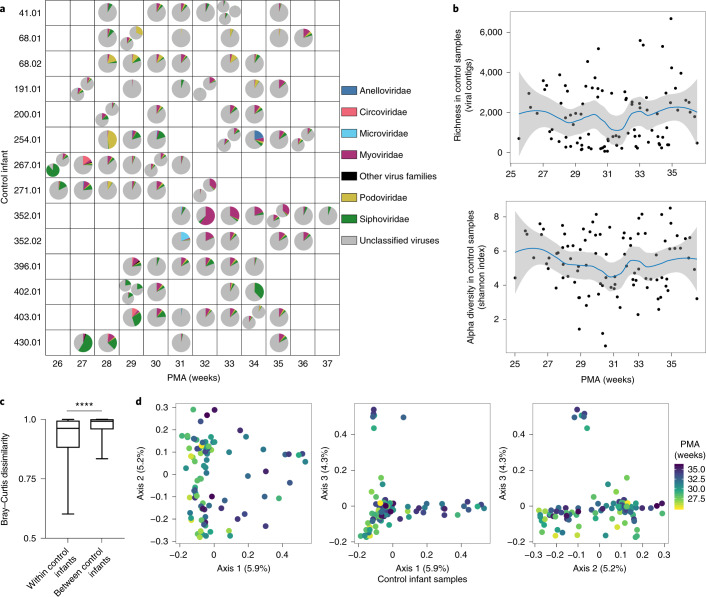
Gut virome in preterm infants who did not develop NEC (controls). **a**, Virus family relative abundance in samples from control infants, grouped by PMA. Multiple pie charts within a square indicate multiple samples from the same infant in one week. **b**, Viral contig richness and alpha diversity (Shannon index) in control samples over time. Trend lines and 95% confidence bands were generated using LOESS smoothing in R. Smoothing level: span = 0.5. **c**, Median weighted Bray–Curtis dissimilarity within individual control infants and between individual control infants, *n* = 14 infants. Box limits, 25th and 75th percentiles; whiskers, 2.5 and 97.5 percentiles. Statistical significance was assessed by two-sided Mann–Whitney *U*-test, *P* < 0.0001. **d**, PCoA of control samples, using weighted Bray–Curtis distance. Statistical significance of PMA (continuous variable) was assessed by PERMANOVA. Samples were colour-coded by PMA.

**Extended Data Fig. 2:**
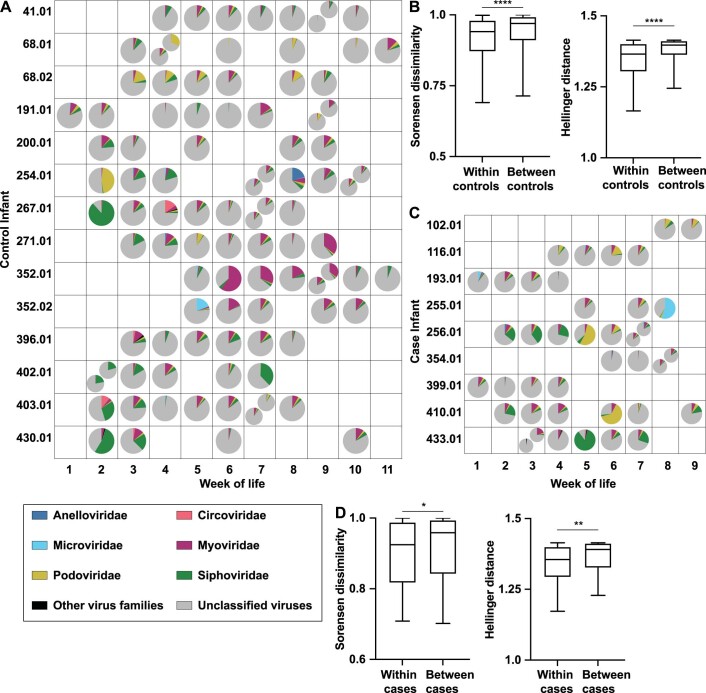
Relative abundance by week of life, Sorensen dissimilarity and Hellinger distance within and between infants. **a**: Virus family relative abundance in control samples from Fig. 1a, grouped by week of life rather than postmenstrual age. Multiple pie charts in the same square indicate multiple samples for an infant in the same week. **b**. Sorensen dissimilarity and Hellinger distance within and between control infants, n = 14 infants. Center line = median; box limits = 25th and 75th percentiles; whiskers = 2.5 and 97.5 percentiles. Statistical significance assessed by two-sided Mann-Whitney test, p < 0.0001. **c**: Virus family relative abundance in case samples from Fig. 2a, grouped by week of life rather than PMA. Multiple pie charts in the same square indicate multiple samples for an infant in the same week. The same colors are used to indicate virus families in A and C. **d**: Sorensen dissimilarity and Hellinger distance within and between case infants, n = 9 infants. Center line = median; box limits = 25th and 75th percentiles; whiskers = 2.5 and 97.5 percentiles. Statistical significance assessed by two-sided Mann-Whitney test, p = 0.04 and p = 0.002, respectively.

A large proportion of the virome of control preterm infants could not be assigned family-level taxonomy (unclassified viruses) (median relative abundance = 85.3%; IQR = 79.8–91.3%) (Fig. 1a and Supplementary Table 5). Viral contigs that could be classified belonged to bacteriophage families including *Myoviridae*, *Podoviridae* and *Siphoviridae*. These families were present in controls at median relative abundances of 5.1% (IQR = 1.7–8.4%), 1.8% (IQR = 0.6–2.6%) and 3.8% (IQR = 1.9–5.0%), respectively. *Microviridae* was highly abundant (19.2%) in a single sample but much less abundant (median = 0.1%; IQR = 0–0.2%) in the remaining 94 control samples. Low-abundant phage families in the stools of control infants included *Gokushovirinae,* *Herelleviridae* and *Tectiviridae*. Some control samples had high relative abundance of eukaryotic virus families *Anelloviridae* (1 sample, 20.7%) and *Circoviridae* (2 samples, 10.4 and 15.0%), while other control samples had considerably lower relative abundances (*Anelloviridae*, 8 samples, IQR = 0.1–0.9%; *Circoviridae*, 31 samples, IQR = 0.01–0.3%). Relative abundances of bacteriophage and eukaryotic virus families varied between control infants in each week of the study, spanning the 26th to the 37th week PMA. Family relative abundance also varied within individuals over time. Grouping samples by week of life, rather than the PMA at which they were obtained, yielded similar variation at each time point (Extended Data Fig. 2a). Contig richness and Shannon diversity varied within and between individuals (Fig. 1b and Supplementary Table 2). After controlling for repeated sampling of individuals by linear mixed modelling, neither richness nor Shannon diversity changed significantly over time (*P* = 0.47 and *P* = 0.61, respectively). Finally, we compared viromes between control preterm infants by examining beta diversity. Median weighted Bray–Curtis dissimilarity, which accounts both for virus presence–absence and virus abundance, was significantly lower within than between infants (Mann–Whitney *U*-test, *P* < 0.0001) (Fig. 1c and Supplementary Table 6). We observed similar results for Sorensen dissimilarity (*P* < 0.0001) and Hellinger distance (*P* < 0.0001) (Extended Data Fig. 2b and Supplementary Tables 7 and 8). Principal coordinates analysis (PCoA) on weighted Bray–Curtis dissimilarity showed substantial overlap of samples obtained at different postmenstrual ages, while permutational multivariate analysis of variance (PERMANOVA) testing showed a significant association with PMA (*P* = 0.05) (Fig. 1d and Supplementary Table 9). Taken together, these results demonstrate high inter- and intraindividual variation in the preterm infant gut virome. However, gut viromes in individual infants were more similar to self than to non-self (other infants) over time, indicating some degree of intra-host stability.

### Preterm NEC case infant viromes vary when compared by age

**Fig. 2:**
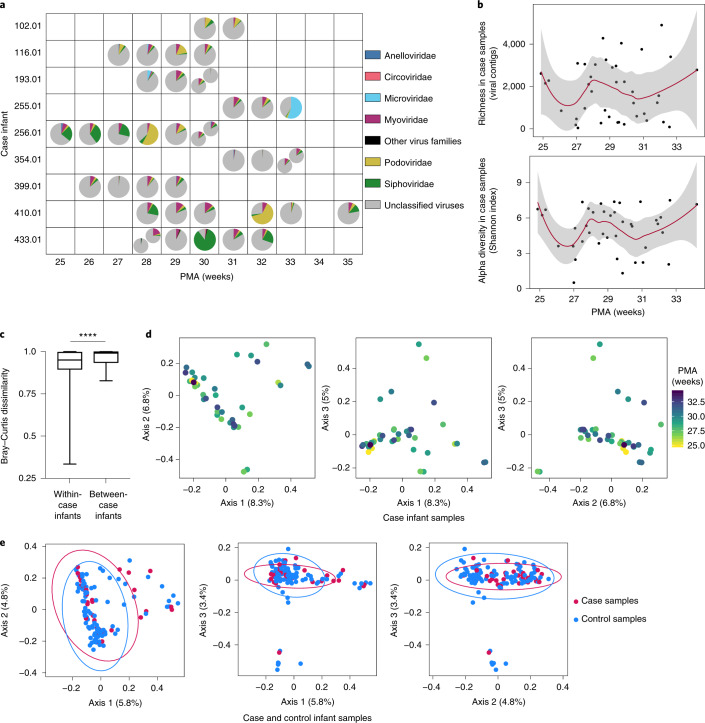
Gut virome over time in infants who developed NEC (cases). **a**, Virus family relative abundance in case samples, grouped by PMA. Multiple pie charts within a square indicate multiple samples from the same infant in one week. **b**, Viral contig richness and Shannon diversity in case samples over time. Trend lines and 95% confidence bands were generated using LOESS smoothing in R, with a span of 0.5. **c**, Median weighted Bray–Curtis dissimilarity within individual case infants and between individual case infants, *n* = 9 infants. Box limits, 25th and 75th percentiles; whiskers, 2.5 and 97.5 percentiles. Statistical significance was assessed by two-sided Mann–Whitney *U*-test, *P* < 0.0001. **d**, PCoA of case samples using weighted Bray–Curtis distance. Statistical significance of PMA (continuous variable) assessed by PERMANOVA. Samples were colour-coded by PMA. **e**, PCoA comparing case and control samples using weighted Bray–Curtis distance. Statistical significance was assessed by PERMANOVA.

We next examined the viromes of the nine case infants who subsequently developed NEC. Median age at NEC onset was 31.1 weeks’ PMA (IQR = 30.0–32.8 weeks); 4 case infants died. As with the control preterm infants, a substantial proportion of case infant viromes consisted of viruses that could not be assigned family-level taxonomy and were categorized as unclassified viruses (median relative abundance = 84.5%; IQR = 75.8–88.2%). Classifiable viral contigs in the case viromes included the bacteriophage families *Myoviridae*, *Podoviridae* and *Siphoviridae*, with median relative abundances of 5.6% (IQR = 1.6–7.4%), 2.7% (1.8–4.1%) and 2.9% (1.7–5.4%), respectively (Fig. 2a and Supplementary Table 5). As in the controls, *Microviridae* were present at high relative abundance (55.4%) in only 1 sample and at lower relative abundances in other samples (median relative abundance = 0.1%; IQR = 0.01–0.2%). Other low relative abundance bacteriophages include *Gokushovirinae,* *Herelleviridae*, and *Tectiviridae*. Several samples contained eukaryotic viruses belonging to the *Anelloviridae* (9 samples, median relative abundance = 0.9%; IQR = 0.2–1.3%) and *Circoviridae* (13 samples, median relative abundance = 0.02%; IQR = 0.01–0.04%) families. We found high variability in virus family proportions at each time point and also within individuals over time. As in the controls, we saw similar variability after grouping samples by week of life (Extended Data Fig. 2c). Shannon diversity and richness varied between individuals and over time (Fig. 2b) but did not change significantly by PMA (linear mixed modelling, *P* = 0.91 and *P* = 0.88, respectively). As with control infants, median weighted Bray–Curtis dissimilarity within individual case infants was significantly less than between individuals (Mann–Whitney *U*-test, *P* < 0.0001) (Fig. 2c and Supplementary Table 6). Results were similar for Sorensen dissimilarity (*P* = 0.04) and Hellinger distance (*P* = 0.002) (Extended Data Fig. 2d and Supplementary Tables 7 and 8). PCoA analysis on weighted Bray–Curtis dissimilarity did not show clustering based on PMA (PERMANOVA, *P* = 0.57) (Fig. 2d and Supplementary Table 9). We next compared virome composition between case and control infants by PCoA (Fig. 2e and Supplementary Table 9). Case and control samples overlapped substantially, while PERMANOVA testing showed no significant difference between the two groups (*P* > 0.99). These results suggest that like the gut viromes of preterm infants without NEC, the gut viromes of infants who subsequently developed NEC vary between and within individuals over time.

### Virome convergence precedes NEC onset

**Fig. 3:**
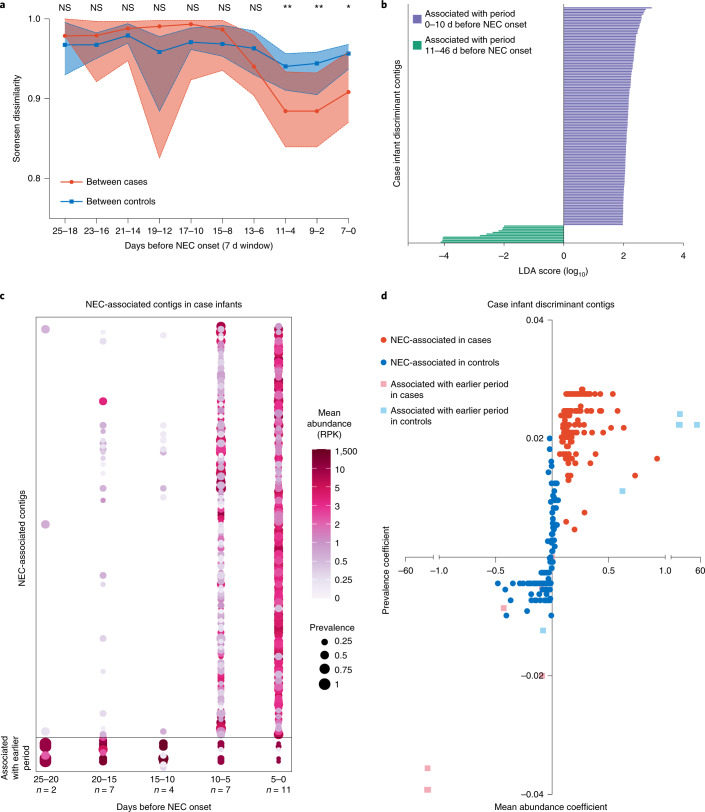
Virome convergence before NEC onset. **a**, Sorensen dissimilarity between cases (red) and between controls (blue) in sliding windows before NEC onset (7 d windows with 2 d steps). Medians with 95% confidence intervals are shown. Statistical significance at each window was assessed by two-sided Mann–Whitney *U*-test. **b**, LEfSe of contigs in case samples. Purple indicates features associated with 0–10 d before NEC. Green indicates features associated with 10–46 d before NEC. **c**, Prevalence and abundance of NEC-associated contigs in cases, in 5 d intervals before NEC. Statistical significance was assessed by Friedman test with Dunn’s multiple comparisons (*P* values in Supplementary Table 13). **d**, For each discriminant contig, linear regression was performed on prevalence and average abundance in 5 d intervals before NEC in cases (red) and controls (blue). Regression coefficients for abundance and prevalence are shown on the *x* and *y* axes, respectively.

**Extended Data Fig. 3:**
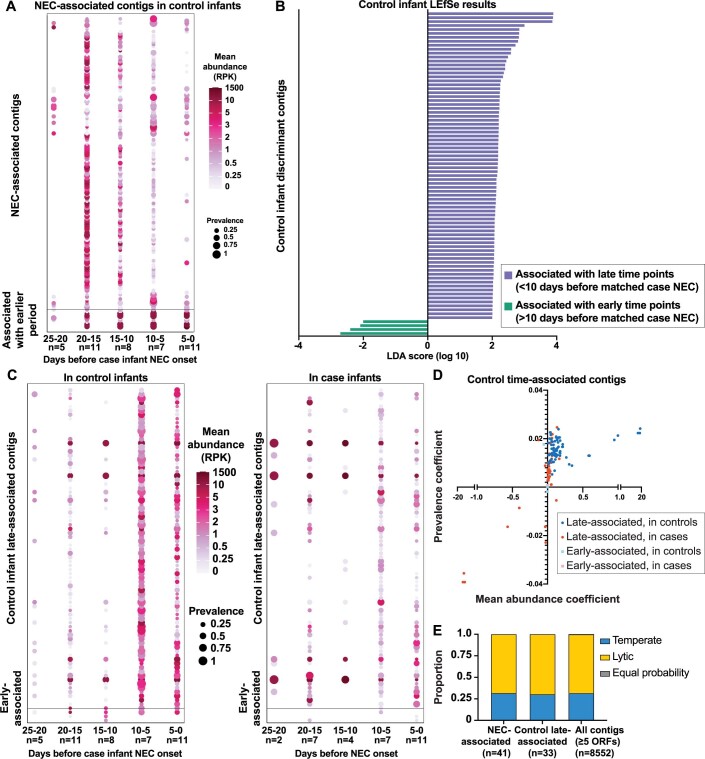
Control infant discriminant contigs and phage lifestyle predictions. **a**: Prevalence and abundance of NEC-associated contigs in control infants, in 5-day intervals prior to respective case NEC onset. Statistical significance assessed by Friedman test with Dunn’s multiple comparisons (p-values in Supplementary Table 14). **b**: Linear discriminant analysis effect size (LEfSe) of contigs in control samples. Purple indicates features associated with 0–10 days prior to case NEC onset. Green indicates features associated with 10–46 days prior to case NEC onset. **c**: Prevalence and abundance of control early- and late-associated contigs in control infants (left) and case infants (right), in 5-day intervals prior to case NEC onset. Statistical significance assessed by Friedman test with Dunn’s multiple comparisons (p-values in Supplementary Tables 17,18). **d**: For each control discriminant contig, linear regression was performed on prevalence and average abundance values in 5-day intervals prior to NEC in cases (red) and controls (blue). Regression coefficients for abundance and prevalence were plotted on the x- and y-axes, respectively. E: Lifestyle predictions (temperate or lytic) for case and control late-associated viral contigs.

We next considered the possibility that virome community dynamics might influence NEC development by examining virome progression in cases relative to the time of NEC onset. We used a sliding 7 d window with steps of 2 d between windows, counting backwards from the day NEC occurred. Sorensen dissimilarity between case infants, which considers virus presence–absence, decreased in sliding windows spanning the 10 d immediately before NEC onset (Fig. 3a, red and Supplementary Table 10). Specifically, viral populations more closely resembled each other in this interval. In contrast, dissimilarity between matched samples among controls was stable during this immediate pre-NEC interval (Fig. 3a, blue and Supplementary Table 10). Between-case dissimilarity was less than between-control dissimilarity at windows spanning 11–4 d, 9–2 d and 7–0 d before NEC (Mann–Whitney *U*-test; *P* = 0.002, *P* = 0.003 and *P* = 0.05, respectively). This suggests that the beta diversity of the gut viromes of case infants begins to converge about 10 d before NEC onset. We then used linear discriminant analysis (LDA) effect size (LEfSe) analysis to identify the viral contigs associated with the 10 d period immediately before NEC onset compared to the immediate antecedent period (that is, 11–46 d before NEC onset). We identified 137 contigs associated with the time period 0–10 d before NEC (NEC-associated contigs), whereas only 11 contigs were associated with the earlier period, that is, 11–46 d before NEC onset (Fig. 3b). Most of these contigs could not be assigned family-level taxonomy, although some belonged to *Myoviridae*, *Podoviridae* and *Siphoviridae* (Supplementary Table 11). We next validated the discriminant analyses by comparing the prevalence and abundance (reads per kilobase (RPK)) of the NEC-associated contigs. We identified NEC-associated contigs in case samples 10–25 d before NEC; however, prevalence and average abundance increased significantly in the 10 d before this event (Fig. 3c and Supplementary Table 12; Friedman test with Dunn’s multiple comparisons, see Supplementary Table 13 for the *P* values). The overall relative abundance of the NEC-associated contigs was low (<20%, data not shown), suggesting that changes in low-abundant viruses are implicated in NEC virome risk convergence. This is consistent with the Sorensen dissimilarity data, which do not consider species abundance. We reasoned that a NEC-associated signature should be progressively enriched closer to NEC onset. Moreover, enrichment should be specific to case infants but not gestational age-matched control infants. Indeed, prevalence and abundance of NEC-associated contigs increased in case infants in relation to controls (Fig. 3d (compare red to blue) and Supplementary Table 12). By contrast, prevalence and abundance of NEC-associated contigs decreased in controls in proximity to case NEC onset (Extended Data Fig. 3a and Supplementary Table 14). To determine if the large number of contigs associated with NEC onset was characteristic of longitudinal virome development, we conducted a similar LEfSe analysis using control samples. Specific contigs were associated with late and early time points in control infants (< or >10 d before their respective case infant’s time of NEC onset (Extended Data Fig. 3b and Supplementary Table 15). Prevalence and abundance of the control late-associated contigs increased significantly over time in controls, whereas in case infants prevalence of these contigs varied and abundance rose slightly (Extended Data Fig. 3c,d and Supplementary Tables 16–18). Notably, the NEC-associated contigs in case infants were different from the late-associated contigs in controls. Of the NEC-associated contigs with at least 5 open reading frames (ORFs), 31.7% were predicted to have temperate lifestyles and 68.3% were predicted to be lytic (Extended Data Fig. 3e). The proportions of predicted lytic and temperate viruses did not differ significantly between NEC-associated contigs, control late-associated contigs and the dataset as a whole (chi-squared test, *P* > 0.9). Taken together, these results indicate that the gut viromes of preterm infants who developed NEC converged in beta diversity before the event and this convergence was driven by enrichment of specific viruses and loss of others. While control infants also gained and lost viral contigs over time, the specific viruses gained and lost differed from those gained and lost in case infants. Furthermore, this turnover was insufficient to drive a substantial change in beta diversity in control infants.

### Bacterial-viral interactions before NEC onset

**Fig. 4:**
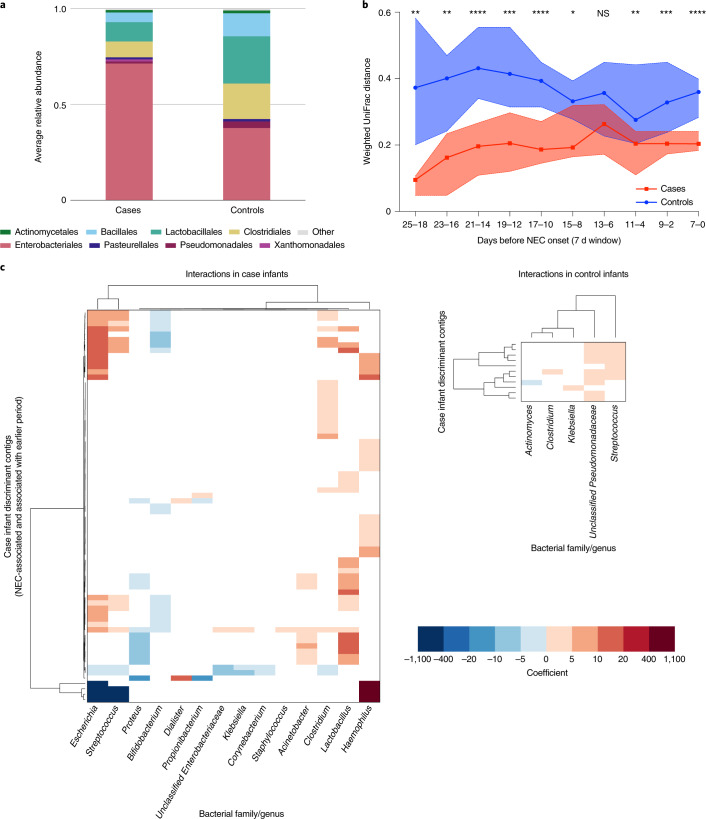
Bacterial microbiome stability and virus-bacteria interactions before NEC onset. **a**, Average relative abundance of bacterial orders in case and control samples in the 25 d preceding case NEC onset. **b**, Weighted UniFrac distance between case samples (red) and between matched control samples (blue) in sliding windows before time of case NEC onset (7 d windows with 2 d steps). Medians with 95% confidence intervals are shown. Statistical significance at each window was assessed by two-sided Mann–Whitney *U*-test. **c**, Significant correlations between case discriminant contigs (NEC-associated and associated with earlier period) and bacterial genera in case (left) and control (right) infants. Dendrograms were ordered based on row and column means. Coefficient refers to the linear regression coefficient (that is, slope).

**Extended Data Fig. 4:**
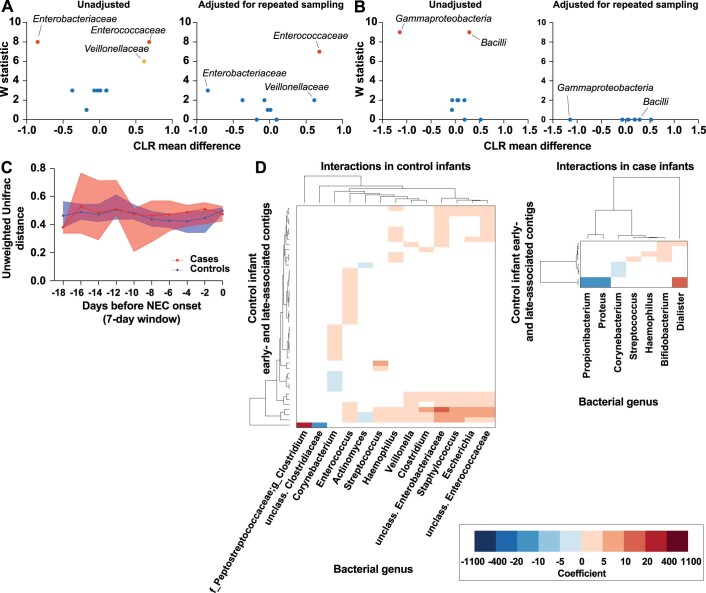
ANCOM-II, unweighted bacterial beta diversity, and bacterial-viral interactions in controls. **a**: ANCOM-II comparing bacterial family abundance in case and control samples, unadjusted (left) and adjusted (right) for repeated sampling. W statistic is the number of significant comparisons between a taxon and the other taxa being tested. CLR mean difference corresponds to effect size. Points in red represent taxa that are differentially abundant when W statistic threshold is set at the 70^th^ percentile. Points in orange represent taxa that are differentially abundant when W statistic threshold is set at the 60^th^ percentile. Points in blue represent taxa that are not differentially abundant. **b**. ANCOM-II comparing bacterial class abundance in case and control samples, unadjusted (left) and adjusted (right) for repeated sampling. W statistic is the number of significant comparisons between a taxon and the other taxa being tested. CLR mean difference corresponds to effect size. Points in red represent taxa that are differentially abundant when W statistic threshold is set at the 70^th^ percentile. Points in blue represent taxa that are not differentially abundant. **c**: Unweighted UniFrac distance between case samples (red) and between matched control samples (blue) in sliding windows prior to NEC onset (7-day windows with 2-day steps). Medians with 95% confidence intervals are shown. Statistical significance at each window assessed by two-sided Mann-Whitney U test. **d**: Significant correlations between control discriminant contigs (late- and early-associated) and bacterial genera in control infants (left) and case infants (right). Dendrograms were ordered based on row and column means.

We next considered the possibility that virome convergence might mirror changes in the bacterial microbiome before NEC onset. Therefore, we used a similar approach to examine bacterial sequencing data^6^ from these samples (that is, in reference to when NEC occurred). Major classes of bacteria found in cases and controls included *Gammaproteobacteria*, *Clostridia* and *Bacilli* (Fig. 4a and Supplementary Table 19). *Enterococcaceae* abundance was significantly different between case and control infants (Extended Data Fig. 4a; ANCOM-II, adjusted for repeat sampling). Differences in *Gammaproteobacteria*, *Bacilli*, *Enterobacteriaceae* and *Veillonellaceae* abundances were not significant when adjusted for repeated sampling (Extended Data Fig. 4a,b). Interestingly, unlike virome beta diversity, bacterial beta diversity in case infants was stable in windows spanning the 25 d before NEC (Fig. 4b and Supplementary Tables 20 and 21). Weighted UniFrac distance during this time was significantly less in case infants than control infants, while unweighted UniFrac distance was not (Fig. 4b, Extended Data Fig. 4c and Supplementary Tables 22 and 23). We did not observe a convergence of the bacterial microbiome in case infants, possibly because case bacterial beta diversity was already low 18–25 d before NEC.

Finally, we investigated the interactions between the virome and bacterial microbiome using linear mixed modelling to identify correlations between viral and bacterial abundance. We found that the NEC-associated contigs were correlated with specific bacterial genera in case infants but did not follow the same correlation pattern in control infants (Fig. 4c, left and Supplementary Table 24). For example, several NEC-associated contigs were positively correlated with *Escherichia* and *Streptococcus*, while many of the contigs associated with >10 d before NEC were negatively correlated with these genera. Correlations between NEC-associated contigs and *Proteus* and *Bifidobacterium* were generally negative. On the other hand, correlations with *Acinetobacter*, *Clostridium*, *Lactobacillus* and *Haemophilus* were generally positive. These specific interactions were absent in control infants (Fig. 4c, right and Supplementary Table 25). We also observed interactions between control late-associated contigs and bacterial genera in control samples (Extended Data Fig. 4d, left and Supplementary Table 26). For example, specific contigs were positively correlated with *Enterococcaceae*, *Escherichia*, *Staphylococcus*, *Enterobacteriaceae*, *Clostridium*, *Veillonella*, *Haemophilus*, *Streptococcus* and *Enterococcus* in control samples. We found relatively few associations between control time-associated contigs and bacterial genera in case samples, except for some positive correlations with *Dialister*, *Bifidobacterium*, *Haemophilus* and *Streptococcus*, and some negative correlations with *Corynebacterium*, *Proteus* and *Propionibacterium* (Extended Data Fig. 4d, right and Supplementary Table 27). Overall, these results indicate that virus-bacteria interactions in case infants who developed NEC differed substantially from control preterm infants who did not develop NEC.

## Discussion

We identified convergence of viral communities and specific viral contigs in the days before NEC onset. The viral signatures of NEC were observed immediately before NEC (beginning at 10 d preceding onset), compared to the bacterial shift observed 25 d before NEC occurred. Detecting patterns of change in the virome before NEC onset could enable early identification of preterm infants at excessive risk of developing NEC.

We found substantial interindividual variation in preterm infant gut viromes (Figs. 1 and 2). Viral family relative abundance, richness and alpha diversity varied between and within individuals over time. We found that within-individual Bray–Curtis dissimilarity was significantly lower than between individuals, suggesting that over time the viromes of individual infants were more similar to self than non-self. This is consistent with previous studies that found substantial interpersonal variation in the adult virome^18,19^. The high proportion of *Microviridae*, *Anelloviridae* and *Circoviridae* in some samples could reflect our use of Φ29 DNA polymerase for viral DNA amplification, which biases towards small circular single-strand DNA viruses^23^.

Interestingly, the gut viromes of preterm infants who developed NEC converged before NEC ensued (Fig. 3). While viruses were associated with specific times before NEC onset in both cases and controls, the specific viruses in each group differed. This indicates that accrual of NEC-associated viruses may be a distinctive feature of the pre-NEC state. For example, virome convergence and shift may alter mucosal immunity. Indeed, bacteriophages have been implicated in mucosal immunity and pathobiology^28–30^. For example, *Escherichia coli, Lactobacillus plantarum* and *Bacteroides thetaiotaomicron* bacteriophages stimulate interferon-γ production through Toll-like receptor 9 signalling independent of bacteria and *E. coli* bacteriophages worsen colitis in mice^30^. *Staphylococcus aureus* and *Pseudomonas aeruginosa* bacteriophages stimulate both pro- and anti-inflammatory gene expression and cytokine production^31,32^. Given the ability of diverse bacteriophages to directly influence mucosal immunity, it is possible that the viruses we identified trigger a cascade that stimulates inflammatory mucosal responses and contributes to NEC pathogenesis. It is also possible that increases in NEC-associated viruses may be a result of bacterial microbiome alterations and mucosal inflammation occurring in the context of bacterial community metabolism before NEC onset. Current data do not permit us to speculate about the mechanistic underpinnings of bacteriophage kinetics, bacterial interactions and the imminent development of NEC. In addition to direct action of bacteriophages on the host, several possibilities are worthy of consideration. These include lysis-independent effects of bacteriophages on bacterial metabolism and expression of effector molecules, lysis-dependent release of host-injurious bacterial molecules and emergence in the infant host of bacterial resistance to bacteriophages^33^.

We found that in the 25 d before NEC onset, the abundance of *Gammaproteobacteria,* *Bacilli*, *Enterococcaceae*, *Enterobacteriaceae* and *Veillonellaceae* differed between case and control samples, as reported previously^6^ (Fig. 4). Note that while that study focused on the class *Negativicutes*, the preponderance of genera in that class were *Veillonella*. However, only the *Enterococcaceae* family was significantly different when adjusted for repeated sampling. These results might be explained by differences in sample size (subset of cohort), analyses pipelines (QIIME 2) and statistical methodologies (ANCOM-II, adjusting for repeated sampling). Unlike virome beta diversity, bacterial beta diversity did not converge. Rather, in the 25 d before NEC onset, weighted UniFrac distance between case samples was low, that is, the bacterial community was highly similar among case infants. During the same time, interactions between bacterial genera and viral contigs differed in the case and control groups. Interestingly, several NEC-associated contigs were positively correlated with *Escherichia* and *Streptococcus*, while several contigs associated with earlier periods were negatively correlated with these genera. The differential correlation of NEC-associated contigs and contigs associated with earlier periods with clinically relevant^6^ bacterial genera may indicate a role for these viruses in NEC development. Although bacteriophage predation on bacterial communities has been implicated in community modulation in experimental systems in mice^25^, soil^34^ and bacteria isolated from the fecal samples of young children^35^, whether the same mechanism contributes to diseases such as NEC will need to be addressed in future studies.

One limitation of this study was that the study population was focused on a single hospital in the United States. It will be important to determine if geographical factors affect the virome in the context of NEC since geography is one factor that can influence the microbiome^36^ and virome^37,38^. It would also be informative to compare data from healthy full-term infants and preterm infants since full-term infants were not studied in this project, although NEC would not be a clinical outcome to which the virome can be related.

This sequential analysis of the gut virome of extremely preterm infants from birth through to near-term (36 weeks’ PMA) provides insight into community membership and dynamics over time and in the weeks preceding NEC. The convergence of beta diversity before NEC onset, driven by enrichment in specific viruses, supports a new line of investigation in the pathogenesis of NEC, a disease that despite intensive investigation is an important source of morbidity and mortality in preterm infants.

## Methods

### Specimens

This study was approved by the Human Research Protection Office of Washington University in St. Louis School of Medicine and Arizona State University Institutional Review Board. Stools were collected prospectively from preterm infants in the NICU at St. Louis Children’s Hospital as part of a larger study on the preterm infant microbiome^6,16,39,40^ (Supplementary Table 1). Infants were eligible if they weighed ≤1,500 g at birth and were expected to survive past the first week of life^6,16^. Written informed consent, including consent to publish, was obtained from the study participants’ families before enrolment. No compensation was provided for the infant stool samples. All infant stools were collected and held briefly at 4 °C, then stored at −80 °C before analysis.

For this study of the preterm virome, specimens were selected from infants who were born at <27 weeks’ gestational age. Infants with NEC were selected who had Bell stage II or higher NEC^41^. We excluded infants with major congenital anomalies, including congenital heart disease, or spontaneous intestinal perforation without radiographic evidence of NEC. One to two control infants were selected for each case, matched by gestational age (±2 weeks) and weight (±200 g) at birth, and availability of sufficient material to perform total nucleic acid (TNA) extraction. Samples included in the analysis were collected during the first three months of life. Samples were selected based on availability of sufficient material for nucleic acid extraction, avoiding consecutive days of life when possible. No statistical analyses were used to predetermine sample size.

We sequenced 138 samples from 23 preterm infants (9 infants with NEC and 14 gestational age-matched controls; 11 males and 12 females) (Table 1, Supplementary Table 2 and Extended Data Fig. 1) but only 135 were included in the final analysis (2 samples were excluded because they contained insufficient reads and 1 was excluded because it was obtained on a day of life that was substantially older than the rest of the samples). For analyses of the virome and microbiome preceding NEC, we counted time backwards from the day of NEC onset for case infants (Extended Data Fig. 1b). For control infants, we counted backwards from the day of life on which their matched case infant was diagnosed with NEC. For the analysis of virome beta diversity preceding NEC (Fig. 3a), paired case and control samples were used: for each case sample, one to two control samples were selected depending on the number of control infants assigned to that case infant. The closest pairings were chosen based on day of life at sample collection (±3.5 d).

### Virome sequencing

Stools were stored at −80 °C until TNA extraction. Stools (approximately 200 mg) were chipped from frozen stock, diluted in PBS in a 1:6 ratio and filtered through a 0.45 μM membrane. TNA was extracted from stool filtrates using the COBAS AmpliPrep Instrument (Roche Diagnostics). DNA was amplified using Φ29 polymerase (GenomiPhi V2 Kit; GE Healthcare), libraries constructed using the Nextera DNA library preparation kit and sequenced on the Illumina MiSeq platform (v.2, 2 × 250 base pairs (bp)) as described elsewhere^20,42^. PBS spiked with Orsay virus RNA generated by in vitro transcription was used as a positive sequencing control. Samples were randomized for sample processing and NGS using a random number generator. Sample processing and NGS were carried out blind to the experimental groups. Subsequent analyses of sequencing data were not performed blind because sample metadata such as infant ID, age and case/control status were essential for statistical analysis.

### Virome analysis

Sequencing reads were quality-filtered with BBTools (v.37.64)^43^, phiX sequences removed, reads mapping to the human genome removed, paired reads merged and reads deduplicated. Contigs were assembled from the reads with phiX sequences removed using metaSPAdes (SPAdes v.3.14.0) (ref. ^44^). A total of 778,612 contigs were assembled from the infant stool samples. Sample and Orsay control contigs were deduplicated separately using CD-HIT-EST v.4.8.1 at minimum 95% identity and 95% overlap^45^. Overlapping contigs were merged using minimus2 (as implemented in AMOS v.3.1.0, https://sourceforge.net/projects/amos/files/amos/) (overlap minimum 95% identity)^46^. Sample and control contigs were then combined into 1 file and filtered by length (minimum length 800 nucleotides). After deduplication and length filtering, 81,873 sample contigs (median length = 1,379 bases; IQR = 998–2,369 bp) remained for analysis. The length-filtered contigs were queried against the Gut Phage^47^ and the Gut Virome databases^48^ using tblastx (minimum e-value 1 × 10^−3^), resulting in 55,002 candidate viral contigs. The quality-filtered, deduplicated reads from the samples and Orsay controls were mapped to the resulting contig database. Contig counts for each sample were normalized by RPK as follows: (79,000/total quality control reads of sample) × (number of reads mapping to each contig). The resulting read counts for each contig were divided by the contig length in kilobases. After normalization, counts smaller than 0.5 were removed to reduce noise. Circular contigs were identified using VirSorter v.1.0.5 (ref. ^49^).

We used the decontam package v.1.4.0 in R v.3.6.1 (refs. ^50,51^) to identify sequencing contaminants by comparing samples to Orsay controls (threshold = 0.1). Contigs identified as contaminants were removed. Candidate viral contigs were queried against the National Center for Biotechnology Information (NCBI) NT database (downloaded February 2018) using megablast; contigs with high percentage identity and query coverage to the human genome were removed (percentage identity and query coverage both ≥90%; either percentage identity or query coverage ≥95%). Two papillomavirus contigs that were traced to contamination during the sequencing run were removed. After decontamination, 40,210 viral contigs remained (median length = 1,562 bp; IQR = 1,054–3,000 bp), of which 692 were circular.

Taxonomy for the viral contigs was assigned based on the taxonomy of the viruses in the Gut Phage and Gut Virome databases. Contig ORFs were predicted using Prodigal v.2.6.3 (ref. ^52^). Phage lifestyles were predicted using PHACTS v.0.3 (ref. ^53^). Lifestyle predictions were only performed for contigs with at least five ORFs and which were not classified as eukaryotic viruses.

### Ecological analysis

Alpha (Shannon index) and beta (Sorensen dissimilarity and weighted Bray–Curtis dissimilarity) diversities were calculated with the vegan package v.2.5-6 in R^54^, using RPK counts and contig presence–absence. Hellinger distance was calculated with the adespatial package^55^ v.0.3-14 in R using log-transformed RPK counts. PCoA was conducted with the phyloseq package v.1.28.0 in R using weighted Bray–Curtis distance on RPK counts. Samples were binned by week (postmenstrual age, PMA) for representation of virus family relative abundance. Family relative abundance and Bray–Curtis dissimilarity were plotted using Prism v.9.1.0 (GraphPad Software). Alpha diversity, richness and PCoA plots were generated with ggplot2 v.3.3.3–3.3.5 in R. Locally estimated scatterplot smoothing (LOESS) regression was used to obtain trend lines and 95% confidence bands.

Matched case and control samples were used to compare between-case and between-control Sorensen dissimilarity as a function of time preceding NEC onset (sliding windows with a window size of 7 d, with 2 d steps between windows). We used LEfSe to identify contigs associated with different times relative to NEC onset^56^. A prevalence threshold of 10% was set for contigs being tested by LEfSe, that is, contigs were only included in the LEfSe analysis if they were found in at least 10% of the samples being analysed. Prevalence and average abundance of selected contigs were compared in cases and controls in 5 d intervals preceding NEC onset, up to 25 d before NEC. All case and control samples in the 25 d before NEC were included. Prevalence was calculated as the percentage of samples within a time block with a hit to a particular contig. Abundance was calculated by averaging all the case or control samples within a given block. Coefficients of linear regression of individual contig prevalence and abundance over time (Fig. 3d and Extended Data Fig. 3d) were obtained using the LINEST function in Microsoft Excel v.16.45.

### Bacterial microbiome analysis

Previously published 454 16S ribosomal RNA gene sequencing data^6^ were downloaded from the NCBI Sequence Read Archive (SRA) for all 40 case samples and the 41 control samples collected in the 25 d preceding case NEC onset. Quality trimming was performed using bbduk (BBTools v.37.64)^43^, followed by denoising using the dada2 plugin in QIIME 2 v.2019.1 (ref. ^57^). Samples were rarefied to a depth of 2,500 reads. Two samples were dropped because of insufficient reads, resulting in 79 samples being used in the final analysis. Alpha (Shannon index) and beta (weighted and unweighted UniFrac distance) diversity were calculated in QIIME 2. Differentially abundant bacteria in cases and controls were identified using the analysis of microbiome composition (ANCOM-II^58^) in R. Correlations between contig and bacterial abundance were determined by linear mixed modelling as implemented in MaAsLin 2 (ref. ^59^). A prevalence threshold of 10% was set for contigs and bacterial genera being analysed with MaAsLin 2, that is, contigs and bacterial genera had to be present in at least 10% of the samples being analysed to be included. Correlations were considered significant if they had a *P* < 0.05 and *q* < 0.25.

### Statistical analyses

#### Metadata variables

Statistical significance for continuous and categorical variables was assessed using the Mann–Whitney *U*-test or Fisher’s exact test, respectively.

#### Virome analysis

Statistical significance of changes in alpha diversity and richness over time were assessed by linear mixed modelling with postmenstrual age as a fixed effect and infant ID as a random effect. Statistical significance for Bray–Curtis dissimilarity (within-individual dissimilarity compared to between-individual dissimilarity) was determined using the Mann–Whitney *U*-test. Statistical significance for PCoA was determined using PERMANOVA, with PMA as a continuous variable and case or control status as a categorical variable. To analyse the time preceding NEC, differences in case or control Sorensen dissimilarity across multiple time windows were assessed using a Kruskal–Wallis test with Dunn’s multiple comparisons. Differences between case and control dissimilarity at each specific window were assessed by the Mann–Whitney *U*-test. Differences in prevalence and abundance of selected contigs in case samples across different time points were compared using a Friedman test with Dunn’s multiple comparisons.

#### Bacterial analysis

Statistical significance of differences in case or control beta diversity across multiple time windows was assessed using a Kruskal–Wallis test with Dunn’s multiple comparisons. Differences between case and control beta diversity at each window were compared using the Mann–Whitney *U*-test.

Mann–Whitney *U*-tests, Kruskal–Wallis tests with Dunn’s multiple comparisons, Friedman tests with Dunn’s multiple comparisons, Fisher’s exact tests and chi-squared tests were performed in Prism. PERMANOVA was performed using the vegan package in R. Mixed linear modelling for virome alpha diversity and richness was performed using the nlme package v.3.1-149 in R^60^. Where appropriate, we chose non-parametric tests that do not assume data to be normally distributed (for example, Mann–Whitney *U*-test, Kruskal–Wallis test). *P* ≤ 0.05 was considered statistically significant. NS, *P* > 0.05, *P ≤ 0.05, **P ≤ 0.01, ***P ≤ 0.001, ****P ≤ 0.0001.

### Reporting Summary

Further information on research design is available in the Nature Research Reporting Summary linked to this article.

## Supplementary information

Reporting Summary

Supplementary Table Excel workbook containing Supplementary Tables 1–27.

## Data Availability

The sequencing data have been deposited with the NCBI SRA (BioProject ID: PRJNA682649). Reads mapping to the human genome were removed from the submitted sequence data.

## Code availability

The code used for the analyses in this study is available at https://github.com/ASU-Lim-Lab/NEC-Virome.
