# Supplementary material for: Longitudinal gut virome analysis identifies specific viral signatures that precede necrotizing enterocolitis onset in preterm infants
Source: Nat Microbiol. 2022 Apr 21;7(5):653–62. doi: 10.1038/s41564-022-01096-x (PMC9064801; doi:10.1038/s41564-022-01096-x)
Supplement: Supplementary file 1 — Reporting Summary [file 41564_2022_1096_MOESM1_ESM.pdf]

nature research

Corresponding author(s): Efrem Lim; Lori Holtz

Last updated by author(s): 02/23/2022

Reporting Summary

Nature Research wishes to improve the reproducibility of the work that we publish. This form provides structure for consistency and transparency in reporting. For further information on Nature Research policies, see our Editorial Policies and the Editorial Policy Checklist.

Statistics

For all statistical analyses, confirm that the following items are present in the figure legend, table legend, main text, or Methods section.

n/a	Confirmed	
☐	☒	The exact sample size (*n*) for each experimental group/condition, given as a discrete number and unit of measurement
☐	☒	A statement on whether measurements were taken from distinct samples or whether the same sample was measured repeatedly
☐	☒	The statistical test(s) used AND whether they are one- or two-sided<br>*Only common tests should be described solely by name; describe more complex techniques in the Methods section.*
☐	☒	A description of all covariates tested
☐	☒	A description of any assumptions or corrections, such as tests of normality and adjustment for multiple comparisons
☐	☒	A full description of the statistical parameters including central tendency (e.g. means) or other basic estimates (e.g. regression coefficient) AND variation (e.g. standard deviation) or associated estimates of uncertainty (e.g. confidence intervals)
☐	☒	For null hypothesis testing, the test statistic (e.g. *F*, *t*, *r*) with confidence intervals, effect sizes, degrees of freedom and *P* value noted<br>*Give P values as exact values whenever suitable.*
☒	☐	For Bayesian analysis, information on the choice of priors and Markov chain Monte Carlo settings
☒	☐	For hierarchical and complex designs, identification of the appropriate level for tests and full reporting of outcomes
☐	☒	Estimates of effect sizes (e.g. Cohen's *d*, Pearson's *r*), indicating how they were calculated

Our web collection on statistics for biologists contains articles on many of the points above.

Software and code

Policy information about availability of computer code

Data collection	REDCap software (7.3.5) was used to collect and manage metadata associated with study participants.
Data analysis	Code used for the analyses in this study is available at https://github.com/ASU-Lim-Lab/NEC-Virome.<br>We used the following software and packages: BBTools (37.64); BLAST+ (2.7.1); R (3.6.1); (decontam (1.4.0); vegan (2.5-6); phyloseq (1.28.0); gplots (3.1.1); ggplot2 (3.3.3 -- 3.3.5); GraphPad Prism (9.1.0); SPAdes (3.14.0); CD-HIT-EST (4.8.1); minimus2; BWA (0.7.17-r1188); VirSorter (1.0.5); lefse (1.0.0); MaAsLin2 (1.0.0); SAMtools (1.7); Bowtie 2 (2.3.5); adespatial (0.3-14); taxonomizr (0.5.3); nlme (3.1-149); QIIME2 (2019.1); Prodigal (2.6.3); PHACTS (0.3); Microsoft Excel (16.45); tidyverse (1.3.1); compositions (2.0-4); ANCOM v2.1

For manuscripts utilizing custom algorithms or software that are central to the research but not yet described in published literature, software must be made available to editors and reviewers. We strongly encourage code deposition in a community repository (e.g. GitHub). See the Nature Research guidelines for submitting code & software for further information.

Data

Policy information about availability of data

All manuscripts must include a data availability statement. This statement should provide the following information, where applicable:

- Accession codes, unique identifiers, or web links for publicly available datasets
- A list of figures that have associated raw data
- A description of any restrictions on data availability

Sequencing data for this study has been deposited to the NCBI Sequence Read Archive under accession number PRJNA682649. Reads mapping to the human genome have been removed from the submitted sequence data.
The Gut Phage Database (Camarillo-Guerrero et al., 2021) used for virome analysis is available at http://ftp.ebi.ac.uk/pub/databases/metagenomics/genome_sets/gut_phage_database/

The Gut Virome Database (Gregory et al, 2020) used for virome analysis is available at https://doi.org/10.25739/12sq-k03
9

Field-specific reporting

Please select the one below that is the best fit for your research. If you are not sure, read the appropriate sections before making your selection.

☒ Life sciences ☐ Behavioural & social sciences ☐ Ecological, evolutionary & environmental sciences

For a reference copy of the document with all sections, see nature.com/documents/nr-reporting-summary-flat.pdf

Life sciences study design

All studies must disclose on these points even when the disclosure is negative.

Sample size	We selected available stool samples from the study participants, collected during the first three months of life. In total, 138 samples were sequenced. No statistical analysis was used to predetermine sample size.
Data exclusions	We excluded two samples from the virome analysis and two samples from the bacterial microbiome analysis because of insufficient sequencing reads. One sample was excluded from analysis because the infant's age at sample collection was substantially older than any other sample. These criteria were not predetermined.
Replication	We repeated the analysis with multiple databases (initial analysis with NCBI RefSeq; final analysis with Gut Phage Database and Gut Virome Database). We observed a similar virome convergence signature with both analyses.
Randomization	Samples were randomized during sample processing and NGS using a random number generator. Allocation to experimental groups (cases and controls) was based on whether infants did or did not develop NEC, and therefore randomization was not applicable.
Blinding	Blinding was used during sample processing and NGS. Blinding was not applicable for the subsequent analysis because metadata such as infant ID, age, and case/control status were necessary for statistical analysis.

Reporting for specific materials, systems and methods

We require information from authors about some types of materials, experimental systems and methods used in many studies. Here, indicate whether each material, system or method listed is relevant to your study. If you are not sure if a list item applies to your research, read the appropriate section before selecting a response.

Materials & experimental systems

n/a	Involved in the study	
☒	☐	Antibodies
☒	☐	Eukaryotic cell lines
☒	☐	Palaeontology and archaeology
☒	☐	Animals and other organisms
☐	☒	Human research participants
☒	☐	Clinical data
☒	☐	Dual use research of concern

Methods

n/a	Involved in the study	
☒	☐	ChIP-seq
☒	☐	Flow cytometry
☒	☐	MRI-based neuroimaging

Human research participants

Policy information about studies involving human research participants

Population characteristics	The participants in this study were preterm infants who developed necrotizing enterocolitis (n=9; 6 male and 3 female) and preterm control infants who did not develop NEC (n=14; 5 male and 9 female). The case and control infants were matched based on gestational age at birth (+/- 2 weeks) and birthweight (+/-200 grams). All infants were born at <27 weeks gestation. Samples were collected longitudinally during the first 3 months of life. Additional cohort characteristics recorded include delivery route, sex, Apgar scores, exposure to human milk during the sampling period and antibiotic exposure during the sampling period.
Recruitment	All premature infants admitted to the neonatal intensive care unit at St. Louis Children's Hospital were considered for study eligibility, and infants who met the eligibility criteria were enrolled if their family provided informed consent. Infants were eligible if they weighed 1500 grams or less at birth and were expected to survive past the first week of life. Infants with congenital heart disease or spontaneous intestinal perforation without radiographic evidence of NEC were excluded.
Ethics oversight	This research was approved by the Human Research Protection Office, Washington University in St. Louis School of Medicine and the Arizona State University Institutional Review Board.

Note that full information on the approval of the study protocol must also be provided in the manuscript.
